# Microbiome Changes in Pregnancy Disorders

**DOI:** 10.3390/antiox12020463

**Published:** 2023-02-12

**Authors:** Luca Giannella, Camilla Grelloni, Dayana Quintili, Alessia Fiorelli, Ramona Montironi, Sonila Alia, Giovanni Delli Carpini, Jacopo Di Giuseppe, Arianna Vignini, Andrea Ciavattini

**Affiliations:** 1 Woman’s Health Sciences Department, Gynecologic Section, Polytechnic University of Marche, Via Filippo Corridoni, 16, 60123 Ancona, Italy; 2 Department of Clinical Sciences, Section of Biochemistry, Biology and Physics, Università Politecnica delle Marche, Via Tronto 10/A, 60126 Ancona, Italy

**Keywords:** microbiome, pregnancy, pregnancy disorders, dysbiosis, hypertensive disorders, gestational diabetes, preterm birth, recurrent miscarriage

## Abstract

The human microbiota comprises all microorganisms, such as bacteria, fungi, and viruses, found within a specific environment that live on our bodies and inside us. The last few years have witnessed an explosion of information related to the role of microbiota changes in health and disease. Even though the gut microbiota is considered the most important in maintaining our health, other regions of the human body, such as the oral cavity, lungs, vagina, and skin, possess their own microbiota. Recent work suggests a correlation between the microbiota present during pregnancy and pregnancy complications. The aim of our literature review was to provide a broad overview of this growing and important topic. We focused on the most significant changes in the microbiota in the four more common obstetric diseases affecting women’s health. Thus, our attention will be focused on hypertensive disorders, gestational diabetes mellitus, preterm birth, and recurrent miscarriage. Pregnancy is a unique period in a woman’s life since the body undergoes different adaptations to provide an optimal environment for fetal growth. Such changes also involve all the microorganisms, which vary in composition and quantity during the three trimesters of gestation. In addition, special attention will be devoted to the potential and fundamental advances in developing clinical applications to prevent and treat those disorders by modulating the microbiota to develop personalized therapies for disease prevention and tailored treatments.

## 1. Introduction

With the term microbiota, we indicate the collection of all microorganisms, such as bacteria, fungi, and viruses, that are found within a specific environment and thus naturally live on our bodies and inside us. They play essential roles in our metabolism and immune and endocrine systems. It is crucial to note that microbiota composition is not static but rapidly evolves. During our lifetime, these microbial populations have been displayed to change, and this happens from infancy to childhood, adulthood, and old age. On the other hand, the microbiome refers to the collection of genomes from all the microorganisms in that specific environment.

Over the past few decades, since the Human Microbiome Project (HMP) launched in 2007 [1], microbiota research has advanced quickly and has become an area of great scientific and public interest.

The exact definition of healthy microbiota has yet to be defined. Still, studies have shown that a healthy body flora or microbial ecosystem can be maintained using probiotics, prebiotics, and symbiotics. Significant advances have been made, and for this reason, we are now aware of the crucial role of the human microbiota in human health and disease [2]. The gut microbiota is considered the most important in maintaining our health since it has several functions, ranging from food fermentation to protection against pathogens, stimulation of the immune system, and vitamin production [3]. While less well recognized than in the gut, the microbiota is also present in other regions of the human body, among which we can list the oral cavity, lungs, vagina, and skin [4,5,6].

Complications during pregnancy frequently occur (generally without any known etiology), are detected in approximately one in every six pregnancies, and cause a danger to maternal and fetal health and survival [7]. Under normal conditions, the microbiome of a newborn is settled through exposure to bacteria both prenatally and postnatally.

Some bacterial infections have been associated with pregnancy complications, although the precise causal mechanisms are still unknown [8]. More recently, different studies have been conducted to find any correlation between the microbiota present during pregnancy and pregnancy complications [9].

In the present comprehensive narrative review, we will provide a broad overview of the most significant changes in the microbiota occurring in the four more common gynecological diseases affecting women’s health. Thus, we will focus on hypertensive disorders, gestational diabetes (GD), pre-term birth, and recurrent miscarriage. Changes in the oral, vaginal, and intestinal microbiome will be analyzed. Other microbiome sites of growing interest are the skin and placenta. However, the skin microbiome has been most studied in newborns, especially preterm deliveries, with not-so-precise results. Furthermore, no significant evidence exists that the maternal skin microbiome correlates with pregnancy disorders [10,11,12,13,14,15,16,17]. Concerning the placental microbiome, the existence of a resident placental microbiota is still controversial [18,19,20,21,22,23]. Several studies postulated that findings were contaminations in either technique or processing [24,25,26,27,28]. Consequently, we focused our analysis on different sites. 

Finally, special attention will be devoted to the potential and fundamental advances in developing clinical applications to prevent and treat such disorders by modulating the microbiota.

## 2. Microbiome Changes during Pregnancy

**Figure 1:**
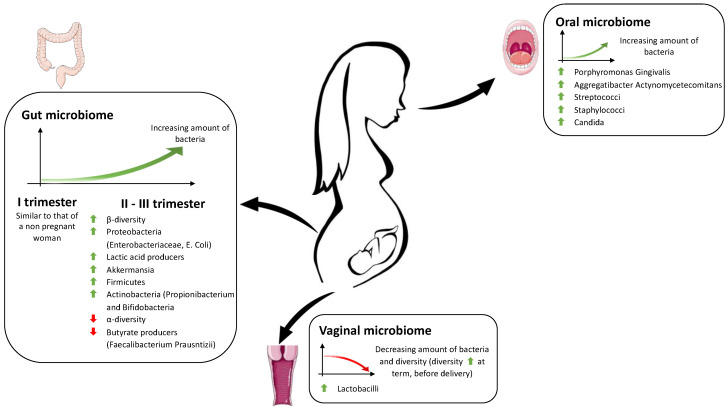
Schematic representation of the main physiological changes in the maternal microbiome during pregnancy. The Figure was partly generated using Servier Medical Art, provided by Servier, licensed under a Creative Commons Attribution 3.0.

Pregnancy is a unique period in a woman’s life, as the body undergoes anatomical, hormonal, metabolic, and immunological adaptations to provide an optimal environment for fetal growth. These changes also involve the microbiome, which varies in composition and quantity during the three trimesters of gestation (Figure 1).

While the role of hormonal changes in pregnancy has been extensively documented [29], maternal microbiome changes, its interaction with the immune system, and its involvement in the pathogenesis of obstetric complications remain to be elucidated.

### 2.1. Oral Microbiome in Pregnancy

The oral microbiome is the second most complex microbial population of the human body. It accounts for more than 700 species residing in different parts of the oral cavity (teeth, gingival sulcus, tongue, cheeks, tonsils, and hard and soft palates) [30].

The resident microbial species primarily belong to 12 phyla: *Actinobacteria*, *Bacteroidetes*, *Chlamydiae*, *Chloroflexi*, *Firmicutes*, *Fusobacteria*, *Gracilibacteria (GN02)*, *Proteobacteria*, *Spirochaetes*, *SR1*, *Synergistetes*, and *Saccharibacteria (TM7)* [31].

The oral microbiome increases in bacterial load during pregnancy while its richness, diversity, and composition remain relatively stable throughout gestation [32,33,34,35]. Fujiwara et al. documented a more significant number of microorganisms in the salivary samples of Japanese pregnant women compared to non-pregnant women [36]. Specifically, they found that significantly more *Porphyromonas gingivalis*, *Aggregatibacter actinomycetemcomitans*, *Streptococci*, *Staphylococci*, and *Candida species* represented in pregnant women, especially during early and middle pregnancy [36]. Another study by Borgo et al. reinforces this concept, showing higher levels of *A. actinomycetemcomitans* in the second and third trimesters compared to non-pregnant women [37]. 

Any alteration of these physiological changes may harm the pregnancy, as it has previously been reported that the prevalence of oral bacteria such as *Campylobacter rectus*, *Fusobacterium nucleatum*, and *Porphyromonas gingivalis* may contribute to the development of adverse obstetric events [38,39,40].

Physiological elevation of sexual steroid hormones during pregnancy, poor health status, and microbiome dysbiosis may increase susceptibility to oral diseases, such as periodontal disease, tooth sensitivity, tooth loss, gingivitis, and gum bleeding [33,41,42]. Periodontitis and gingival inflammation, combined with a dysbiotic oral cavity, have been associated with adverse obstetrical outcomes, such as low birth weight, premature birth [43,44], preeclampsia [41], and miscarriage [45].

### 2.2. Vaginal Microbiome in Pregnancy

The vaginal microbiome varies during a woman’s reproductive life [46]. Bacteria from *Lactobacillus species* predominantly colonize a healthy, non-pregnant vaginal tract. *Lactobacillus* mainly has a protective role against pathogenic bacteria through the induction of a low vaginal pH (3.8–4.4) and the production of lactic acid, bacteriocins, and hydrogen peroxide [47].

Ethnicity is strongly associated with the composition of the vaginal microbiome; in particular, the most remarkable differences were found between the European and African populations [46]. 

Ravel et al. analyzed 396 vaginal samples from four ethnic groups of women (Caucasian, Afro-American, Hispanic, and Asian) using pyrosequencing of barcoded 16S rRNA.

They identified five main groups called community state types (CSTs): CST I (dominated by *L. crispatus*), CST II (dominated by *L. gasseri*), CST III (dominated by *L. iners*), CST IV (lower percentage of *Lactobacillus*, with an increase of anaerobic microorganisms among which *Prevotella*, *Dialister*, *Atopobium*, *Gardnerella*, *Megasphaera*, *Peptoniphilus*, *Sneathia*, *Eggerthella*, *Aerococcus*, *Finegoldia*, and *Mobiluncus*) and CST V (dominated by *L. jensenii*). CST I showed the lowest pH (4.0 ± 0.3), whereas CST IV presented the highest median pH (5.3 ± 0.6). Moreover, the relationship between ethnic background and vaginal bacterial community composition was investigated. Notably, CSTs dominated by *Lactobacilli* (CST I, II, III, and V) were found in 80.2% and 89.7% of Asian and Caucasian women and only in 59.6% and 61.9% of Hispanic and Afro-American women, respectively.

CST IV was much more frequent in Hispanic (34.3%) and Afro-American (38.9%) ethnic groups than in Asian (17.6%) and Caucasian (9.3%) ones. Women belonging to CST IV (which is rich in facultative anaerobic bacteria and shows a lack of *Lactobacilli*) presented a higher rate of short cervix detection in pregnancy and a higher risk of preterm birth.

The reasons for these differences are still unclear and need further investigation. Genetic factors and human habits, including personal hygiene, contraceptive methods, and sexual behaviors, are supposed to be the main influencing factors [46].

During pregnancy, the vaginal microbiota decreases in richness and diversity, and *Lactobacillus species* play a dominant role. It is assumed that this microbiome stability is related to the higher levels of estrogen concentration, the absence of menses, and the modification of cervical and vaginal fluid.

In uncomplicated gestations, the taxonomic composition of the vaginal microbiota remains stable, except during the term of pregnancy, before delivery, when an increased microbial diversity takes place. Through this increase in final diversity, the vaginal microbiome becomes similar to that of the non-pregnant state and is thought to act as a trigger for the onset of labor [48].

### 2.3. Gut Microbiome in Pregnancy

The intestinal microbiota, with its multiple functions, has a significant impact on human health and is currently the main topic of interest for numerous researchers. The gut microbiome influences the host’s metabolism through several mechanisms and plays a crucial role in energy extraction [49].

Fiber-fermenting gut bacteria, such as *Bacteroides*, are essential for indigestible polysaccharides metabolization. They regulate fat storage and produce crucial nutrients.

The intestinal microbiome provides food substances, such as vitamins and minerals, and carries undigested food through the last tract of the digestive system. It also detoxifies and removes xenobiotics from the organism. It helps maintain the integrity of the gut epithelium and acts as a barrier, preventing pathogenic microbes from entering the bloodstream [50].

When a pathogenic microorganism tries to penetrate the bloodstream through the gut wall, the bacterial lipopolysaccharides (LPSs) are recognized by Toll-Like 4 (TLR-4) receptors located on the membranes of the intestinal epithelium, causing systemic inflammation [50].

In the human organism, *Firmicutes*, *Bacteroidetes*, *Actinobacteria*, and *Proteobacteria* constitute 70–90% of all bacteria inhabiting the gastrointestinal tract [51,52], with an evident prevalence of *Firmicutes* and *Bacteroides*. Female gut microbiomes can be classified into different classes called “enterotypes.” Nowadays, three kinds of enterotypes are recognized: enterotype I, characterized by the presence of *Bacteroides*; enterotype II, represented by *Prevotella*; and enterotype III, dominated by *Ruminococcus*. The three enterotypes are probably influenced by diet and perform specific functions, such as energy extraction [53,54]. The European diet, rich in animal protein and lipids, is associated with enterotype I (*Bacteroides*), which produces energy mostly from proteins and carbohydrates [55]. In a healthy pregnancy, the gut microbiome physiologically changes in quantity, composition, and functioning to promote metabolic and immunological changes beneficial for maternal and fetal health [56]. In normal pregnancies, during the first trimester, the intestinal microbiota resembles that of a healthy non-pregnant woman, with a predominance of *Firmicutes* (especially *Clostridiales* and *Faecalibacterium prausnitzii*) over *Bacteroides* [57,58,59,60]. Subsequently, the gut microbiome increases, whereas the composition changes dramatically over gestation [61]. From the second to the third trimester, a progressive reduction in α-diversity (intra-individual inter-species diversity) and an increase in β-diversity (between-subject diversity) occurs [62]. These findings during pregnancy could be due to progressive weight gain (within the normal range) and insulin resistance considered beneficial for fetal growth. These changes in diversity can be considered physiological adaptations [60].

Butyrate-producing bacteria, known for anti-inflammatory properties, decrease, whereas the amount of *Bifidobacteria*, *Proteobacteria*, and lactic acid-producing bacteria grow [57,60,61,63].

As gestation progresses, the gut microbiome is gradually enriched with bacteria that promote weight gain, production and storage of energy, and insulin resistance, which are essential for fetal growth and future breastfeeding [64]. Indeed, the growing presence of *Akkermasia*, *Bifidobacterium*, and *Firmicutes* is associated with rising energy storage [65]. This diabetogenic condition is supported by an exciting study that tested the transplantation of third-trimester gut bacteria into germ-free mice, inducing metabolic changes similar to gestational diabetes [57]. The progressive enrichment in *Actinobacteria* (especially *Propionibacterium*) and *Proteobacteria* (*Enterobacteriaceae*, *Escherichia coli*) could be beneficial for the protection of the maternal–fetal complex from external infections [57,65,66]. During the third trimester, a significant reduction in bacteria producing short-chain fatty acids (for example, *Faecalibacterium prausnitzii*) is observed, with lower butyrate production. This metabolic change is associated with low-grade inflammation, reduced insulin sensitivity, and increased intestinal absorption of essential elements [57,60,64]. 

## 3. Microbiome Changes in Pregnancy Disorders

### 3.1. Hypertensive Disorders

Hypertensive pregnancy disorders (HPDs) complicate up to 10% of pregnancies worldwide; if untreated, these conditions can cause adverse effects on both the mother and child, such as preeclampsia, fetal growth restriction (FGR), and preterm birth [67,68]. Gestational hypertension is defined as pressure values persistently ≥140/90 mmHg in outpatient controls after the 20th week of gestation in normotensive women before pregnancy [69]. 

Preeclampsia (PE) is one of the most common causes of morbidity and mortality for mothers and their offspring. This condition is characterized by signs and symptoms of organ damage, such as proteinuria, renal insufficiency, thrombocytopenia, hepatic dysfunction, and pulmonary edema [70,71]. 

The pathogenesis of PE is still unclear, but some risk factors have been identified in high-risk women, including pregestational obesity, chronic hypertension, family history, and a previous pregnancy complicated by preeclampsia.

One of the most reliable theories on the pathogenesis of preeclampsia assumes that a chronic inflammatory state can influence the process of placentation, hindering the physiological changes that allow the correct function of the organ. Abnormal placentation is associated with uteroplacental ischemia that begins during the first trimester and can lead to a hypertensive and multi-organ failure state called “preeclamptic syndrome” [72].

Recent technologies, such as Next Generation Sequencing (NGS), revealed microbiome modifications during pregnancy, mainly when complications occur. Any alteration or shift in the microbiome balance (dysbiosis) could be involved in inflammatory processes that potentially contribute to adverse pregnancy outcomes [72].

#### 3.1.1. Oral Microbiome and Preeclampsia

The relationship between the oral microbiome and adverse pregnancy outcomes is unclear, but some theories have been proposed.

The physiologically increased incidence of periodontitis, gingivitis, leakiness of the oral cavity, and its tendency to bleed during pregnancy can promote a direct translocation of oral bacteria into the maternal bloodstream, resulting in a transient bacteremia that can reach the fetoplacental unit [73]. Transient bacteremia can occur during routine procedures, such as tooth brushing. Indeed, in patients with periodontal disease, the number of oral bacteria detected in the vascular system increases from two- to tenfold compared to healthy controls [74].

Once in the bloodstream, adhesion proteins expressed on the surface of the oral microbes can bind to the placental cell receptors and trigger a downstream inflammatory response [33,75,76].

Another possibility supposes that the systemic dissemination of endotoxins and/or inflammatory mediators could be carried from the unhealthy oral cavity to the fetoplacental unit [19].

One primary interaction between the oral microbiota and hypertensive gestational disorders is associated with the production of nitrogen derivatives, especially nitric oxide (NO). NO, produced from L-arginine through nitric oxide synthases (NOSs), is implicated in vascular processes, especially vasodilatation and tissue protection. NO comes from vegetable sources, such as spinach, lettuce, or beetroot roots. NO aberrant pathways are involved in chronic cardiovascular disorders, including hypertension [77]. 

The oral microbiome can “recycle” nitrate from the blood through the enterosalivary pathway to extend NO bioavailability. After being oxidated and removed from plasma, nitrate is concentrated in the salivary glands. Once in the mouth, it is reduced by selected oral bacteria to nitrite, utilizing the nitrate reductase enzymes. These reducing bacteria are more concentrated on the tongue’s surface [78].

After swallowing, nitrite can be converted into NO by bacterial nitrite reductase enzymes in the gastrointestinal tract. It has various roles, including maintaining the gastric epithelium mucus barrier and mediating gastric blood flow. These reactions can facilitate the production of nitro-fatty acids, such as NO2-conjugated linoleic acid, that are subsequently reintroduced into the plasma [79].

*Neisseria*, *Veillonella*, *Haemophilus*, *Porphyromonas*, *Fusobacterium*, *Prevotella*, *Leptorichia*, *Brevibacillus*, and *Granulicatella* have been identified as the reducing bacteria of the oral cavity using 16-RNA sequencing [80]. Disruption of the oral microbiota, such as mouthwash, has been shown to correlate with a reduction in plasma and salivary nitrite. This reduction is associated mainly with increased stiffness of the smooth muscles and vessels, which could lead to hypertension [81].

Previous research identified a positive correlation between periodontal disease and preeclampsia during pregnancy and an association between maternal oral health and various adverse pregnancy and birth outcomes, early childhood caries, and other chronic diseases. These findings indicate the oral microbiota’s complex and multi-faceted role in health and disease, including during pregnancy [82].

#### 3.1.2. Gut Microbiome and Preeclampsia

The intestinal microbiota acts as a protective agent against many potentially dangerous agents; it can increase energy intake to enable protein synthesis, thus changing free fatty acids, bile acids, and LPSs to help maintain the membranes’ integrity [83]. 

Several metabolic, immune, and hormonal changes occur during pregnancy and strongly influence the fetus’s development. 

Obesity is associated with a specific microbiota composition during gestation, with higher levels of *Bacteroides* and *Staphylococcus* compared to women with a healthy weight [55]. Moreover, an obese woman can present altered levels of proinflammatory cytokines, decreased decidual uterine natural killer cells, and reduced production of proangiogenic factors. Several studies support the hypothesis that alterations (dysbiosis) in the gut microbiota during early pregnancy could increase the risk of gestational diabetes and hypertension, mainly if associated with obesity [84].

Lv et al. found a significant association between alterations in gut microbiota (dysbiosis) and preeclampsia (PE). They described how the gut microbiota composition in patients with PE significantly differs from that in healthy pregnant women. They identified that bacteria associated with PE were associated with other morbidities, such as obesity, glucose metabolic disorders, proinflammatory states, and intestinal barrier dysfunction. In addition, these microorganisms influenced some host immune parameters, including interleukin-6 (IL-6) and lipopolysaccharide (LPS), the major component of the outer membrane of Gram-negative bacteria. These findings suggest that a pathological gut microbiota during early pregnancy, due to an altered maternal immune system and increased proinflammatory cytokines, may be involved in developing pregnancy-related complications such as PE [85]. Huang et al. showed a significant reduction in the abundance of *Prevotella*, *Porphyromonas*, *Varibaculum*, and *Lactobacillus* in the gut microbiome of women with preeclampsia compared to healthy pregnant women [86]. *Prevotella* is implicated in producing short-chain fatty acids (SCFAs), such as butyrate, which lower maternal blood pressure during pregnancy [87]. Butyrate is the primary energy source for cells that constitute the intestinal epithelium and is involved in T lymphocyte differentiation. Several studies showed a protective effect of butyrate on the occurrence of preeclampsia by inhibiting the synthesis of the plasminogen activator-1 inhibitor (PAI-1), which causes a reduction in vasoconstriction and a decreased secretion of nitric oxide (NO), damaging the vascular endothelium [88,89,90].

Previous studies showed an inverse correlation between the number of *Lactobacillus* and the incidence of arterial hypertension in patients with preeclampsia. The study focused on toxins produced by *Lactobacillus OTU255* and *OTU784*. It revealed that *OTU255* was significantly reduced in the group of individuals with preeclampsia, while *OTU784* had decreased considerably in patients with abnormal placental growth. These results highlighted the importance of changes in the microorganisms colonizing the gastrointestinal tract in the etiology of both preeclampsia and abnormal development of the placenta during pregnancy [86,91].

Further studies reported an increased presence of pathogenic microorganisms, particularly *Bulleidia moorei* and *Clostridium perfringens*, and a lower number of *Coprococcus catus* [92].

*Clostridium perfringens* lives in the large intestine and is involved in the metabolism of carbohydrates and proteins. In some circumstances, it can lead to septic shock and affect the cardiovascular system. This bacterium secretes 16 toxins, which can increase blood pressure and lead to disturbances in blood coagulation. They can reduce the speed of blood transport in the body, with a consequently higher risk of vascular diseases. Beta-toxins can lead to necrotizing enterocolitis and the narrowing of blood vessels, which increases blood pressure [93,94]. Liu et al. deduced that an increase in *Clostridium perfringens* might predispose pregnant women to preeclampsia through its toxins and the interactions between this bacterium and other microbes living in the human intestine [92].

The relationship between gut bacteria and hypertensive disorders in pregnancy is still to be elucidated, given the limited and only recent studies present in the literature to date.

**Figure 2:**
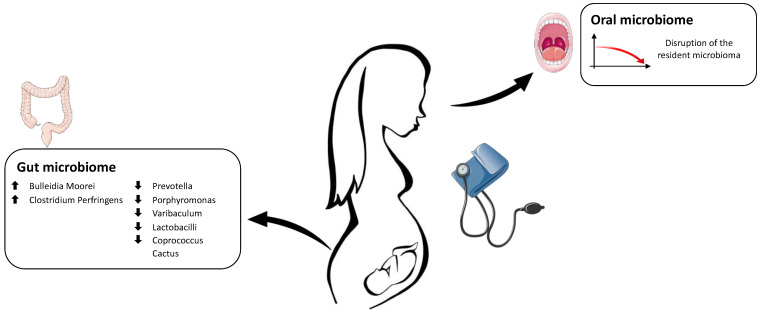
Schematic representation of the main alterations in the maternal microbiome in pregnancies complicated by hypertensive disorders. The Figure was partly generated using Servier Medical Art, provided by Servier, licensed under a Creative Commons Attribution 3.0 unported license.

According to the various anatomical sites, the microbiome changes in pregnancy disorders are represented in Figure 2. 

### 3.2. Gestational Diabetes (GD)

Diabetes is a chronic metabolic disease spread worldwide, especially in low- and middle-income countries. The WHO estimates that there are at least 422 million diabetic people worldwide; without effective interventions, this number is expected to grow to 570 million in 2025 [95,96]. 

The most common is type 2 diabetes (T2D) which usually occurs in adults (mean age 55 to 59 years of age). During the last 30 years, the prevalence of T2D has increased throughout countries of all income levels [95,97]. The International Diabetes Federation (IDF) predicts that, by 2045, T2D patients will increase to 700 million, and, consequently, the social and economic costs associated with the diabetic condition will rise. These numbers were provided before the advent of the COVID-19 pandemic, which worsened the population’s lifestyle, aggravating the estimates [98].

Type 1 diabetes (T1D) is a chronic, multifactorial, autoimmune condition mainly affecting young people. It requires careful management to avoid life-threatening long-term complications. The incidence and prevalence of type 1 diabetes are increasing worldwide, probably influenced by other predisposing factors in addition to genetics [99].

GD is the most common metabolic disorder of pregnancy, with an incidence ranging from 1.8 to 2.2% [100]. The American Diabetes Association (ADA) defines GD as diabetes diagnosed in the second and third trimesters of pregnancy that is not overt diabetes before gestation or in the early stage of pregnancy [101].

Firstly, GD can lead to maternal complications during pregnancy, such as increased rates of preeclampsia, preterm delivery, polyhydramnios, shoulder dystocia, cesarean section, instrumental delivery, postpartum hemorrhage, and perineal lacerations. Secondly, the offspring of mothers with hyperglycemia have an increased incidence of macrosomia, fetal/neonatal death, malformations, prematurity, neonatal jaundice, respiratory distress syndrome, admission to neonatal intensive care unit, and low Apgar score [102,103,104]. 

Among women with a history of GD, there is a lifetime risk of 60% of developing T2D [105]. In the short term (6–12 weeks after delivery), this percentage is around 4% [106]. Moreover, these patients will have a 2-fold higher risk of developing cardiovascular diseases. Newborns from diabetic pregnancies have a two- to eight-fold increased risk of developing obesity and T2D in the early years of life [107,108,109].

#### 3.2.1. Gut Microbiome Changes in Patients with GD

In recent years, there has been growing interest in microbial composition differences between healthy pregnancies and pregnancies with GD, but these are data-limited and still discordant. 

The intestinal microbiome modulates insulin resistance and the inflammatory response, and dysbiosis can be associated with metabolic diseases [110]. However, researchers still need to determine its exact role in GD development.

It is hypothesized that dysbiosis can lead to metabolic diseases through several mechanisms: abnormal gut permeability, increasing absorption of lipopolysaccharide (LPS), aberrant production of short-chain fatty acid, altered conversion of primary bile acids, and expanded production of bacterial toxic substances (e.g., trimethylamine N-oxide) [111,112].

These abnormal mechanisms can cause the activation of inflammatory and autoimmune pathways in the body, stimulate the endocannabinoid system, alter the secretion of intestinal peptides, inhibit insulin signaling, and increase the extraction and storage of energy [113,114].

During pregnancy, the number of gut Gram-negative bacteria expands, and the number of LPS, forming most of their cell wall, increases. This phenomenon affects the integrity of the intestinal epithelium and promotes the infiltration of macrophages and the production of pro-inflammatory cytokines, creating a systemic inflammation state called “metabolic endotoxemia” [50,115,116,117]. In addition, physiological dysbiosis occurs during pregnancy, promoting weight gain, inflammatory cytokines circulation, and insulin resistance [118].

It is hypothesized that a dysbiotic intestine can drive epigenetic alterations in maternal DNA, and in that of the newborn, in a “diabetogenic” and “obesogenic” way through its metabolites, such as LPS, folate, the B vitamins, and enzymes (methyltransferases, acetyltransferases, deacetylases, for instance) [56,119,120].

These epigenetic processes change gene expression without modifying the nucleotide sequence but by exploiting DNA methylation, histone modifications, RNA noncoding regulation, and ATP-dependent chromatin remodeling processes [56].

In a pilot study, Kumar et al. found a strong association between the blood DNA methylation pattern and altered gut microbiota in pregnant women. Among abnormally methylated genes, several were known to be associated with cardiovascular diseases, lipidic dysmetabolism, obesity, and inflammatory status [121].

Specifically, the enteric *Firmicutes* species are known to be associated with the development of obesity and metabolic syndrome. Still, it might promote epigenetic modification by aberrant production of folate and butyrate [121,122], both in the mother and the offspring [123].

These studies suggest that epigenetic changes could be passed on to offspring and future generations, increasing the incidence of obesity, diabetes, and inflammatory diseases. 

Significant differences in the gut microbiome composition patterns were detected in each trimester of pregnancy between normoglycemic and GD pregnancies.

A Finnish study found a reduced microbial richness but no differences in the species composition in the first gestational trimester in women who were subsequently diagnosed with GD. Other research also suggested that the relative abundance of the *Rominococcaceae* family during the initial periods of pregnancy could be related to a future onset of GD [124].

During the first and second trimesters of pregnancy, Zheng et al. reported a lack of dynamic physiological changes in the gut microbiome of a GD group and a consistent decrease in *Coprococcus* and *Streptococcus* associated with a relevant presence of *Megasphera* and *Eggertella* [125]. Hu et al. demonstrated an overrepresentation of *Enterobacteriaceae*, *Ruminococcaceae* spp., and *Veillonellaceae* spp. in a group of pregnant women at 6–15 and 24–28 gestational weeks that were subsequently diagnosed with GD [126]. 

In agreement with previous literature, two recent studies reported that, during the third trimester, the number of *Firmicutes* rises in GD patients, while the *Bacteroides* amount decreases, with a higher *Firmicutes/Bacteroides* ratio (*F/B*), compared to the control group [58,127]. Koren et al. documented a significantly expanded β-diversity and an enrichment in *Actinobacteria* and *Proteobacteria* quantity during third-trimester cases with GD [57].

Crusell et al. found that patients with GD had an altered gut microbiota composition in the last trimester of gestation, resembling the aberrant microbiota of non-pregnant individuals with T2D. This study identified *Actinobacteria* at the phylum level and *Collinsella*, *Rothia*, *Actinomyces*, and *Desulfovibrio* at the genera level as possible biomarkers of GD [128]. Moreover, a decreasing quantity of *Roseburia* and *Fecalibacterium prausnitzii* is documented in late pregnancy with GD. The lack of these butyrate-producing bacteria contributes to the inflammatory state and the insulin-resistant metabolism typical of gestation [57,58,129].

Ferrocino et al. analyzed the fecal microbiome of pregnant women with GD between 24 and 38 weeks. At the term of gestation, the microbiota α-diversity and the amount of *Firmicutes* significantly increased, while *Bacteroidetes* and *Actinobacteria* were reduced. Patients adhering to the diet had a better metabolic and inflammatory profile and a significant decrease in *Bacteroides*, showing that gut microbiota might be modulated by prevention strategies [130].

An Italian pilot study on women in the early stages of the third trimester observed a rich quantity of *Bacteroides caccae*, *Bacteroides massiliensis*, and *Bacteroides thetaiotaomicron*, and a reduced amount of Bacteroides vulgatus, Eubacterium eligens, Lactobacillus rogosae, and *Prevotella copri* in GD patients [131].

Several studies focused on the differences in microflora composition between mothers with GD and normoglycemic mothers. GD patients showed an increased presence of *Collinsella*, *Rothia*, *Desulfovibrio*, *Parabacteroides d.*, *Klebsiella v.*, *Ruminococcus*, *Prevotella*, *Lachnospiraceae*, *Phascolarctobacterium*, and *Christensenellaceae*, with a reduced gut richness of *Akkermansia*, *Methanobrevibacter*, *Roseburia*, *Alistipes*, *Bifidobacterium*, and *Eubacterium* [58,128,130,132].

In two recent studies concerning the puerperium, women with previous GD showed a more extraordinary richness of *Prevotellaceae*, *Collinsella*, *Olensella*, and *Clostridium* 3–16 months after delivery, and reduced amounts of *Firmicutes*, *Fusobacterium*, *Fusobacteriaceae*, and *Ruminococcus* [128,133]. Another study, comparing the microbiome of patients with GD in the first trimester and during the puerperium found that *Eisenbergiella*, *Tyzzerella 4*, and *Lachnospiraceae NK4A136* were still abundant in the GD group 42 days postpartum. In contrast, *Parabacteroides*, *Megasphaera*, and *Eubacterium eligens* groups remained prevalent in the controls [134]. 

Hasan et al. did not find differences in the gut microbiome between women with previous GD and women with an earlier normoglycemic pregnancy five years after childbirth. This finding would exclude that the future development of a T2D depends on an altered microbiome in women with previous GD [135].

Recent research suggests that dysbiosis characterizing women with GD might be vertically transmitted to the baby during pregnancy, not only at birth but over the three trimesters of gestation. Indeed, multiple sample types of maternal and neonatal microbiota shared the population of *Prevotella*, *Streptococcus*, *Bacteroides*, and *Lactobacillus* [84].

This finding testifies that the prevention of vertical transmission must be implemented during the initial stages of pregnancy and that further studies are needed to understand the early-life microbiome formation and colonization mechanisms.

The role of gut bacteria as causal mediators of GD in pregnancy is still to be elucidated, given the limited and only recent studies present in the literature to date.

#### 3.2.2. Vaginal and Oral Changes in Patients with GD

In GD, previous studies documented an increase in the circulation of inflammatory cytokines and vaginal dysbiosis, with an abundance of pathogenic bacteria [136].

Nevertheless, there is still a lack of studies regarding vaginal and oral dysbiosis in women with GD. 

Cortez et al. recently sampled the vaginal microbiome of women with GDM and compared it to that of normoglycemic pregnancies. In order of frequency, the most abundant species in both groups were *Firmicutes*, *Actinobacteria*, and *Proteobacteria*. The authors reported that the phyla *Firmicutes* and *Proteobacteria* were more abundant in diabetic mothers, while *Actinobacteria* were prevalent in healthy mothers without statistical significance. Generally, women with GD showed a significantly higher abundance of *Bacteroides*, *Veillonella*, *Klebsiella*, *Escherichia*, *Shigella*, *Enterococcus*, and *Enterobacter*. In contrast, the control group presented significantly higher levels of *Varibaculum*, *Prevotella*, *Porphyromonas*, and *Ezakiella*. Moreover, these authors did not find significant differences between the two groups’ oral microbiome composition at the species and general levels [58].

Literature concerning this topic is limited, and further studies will be needed to define whether the vaginal and oral microbiome play a role in developing GD. 

**Figure 3:**
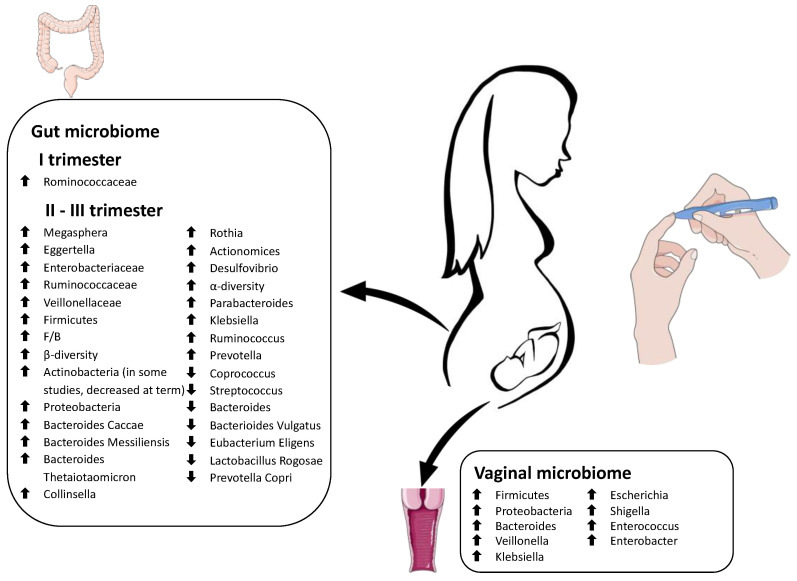
Schematic representation of the main alterations in the maternal microbiome in pregnancies complicated by gestational diabetes. The Figure was partly generated using Servier Medical Art, provided by Servier, licensed under a Creative Commons Attribution 3.0 unported license.

Figure 3 summarizes the main alterations in the maternal microbiome in pregnancies complicated by gestational diabetes.

### 3.3. Preterm Birth

The World Health Organization data shows that preterm birth (PTB) is a common obstetric complication worldwide, affecting 15 million babies yearly. It is defined as the birth of infants before 37 weeks of pregnancy is completed. Globally, complications related to PTB are the leading cause of death among children under five years of age; therefore, the reduction in prematurity rates represents a global challenge for today [137].

The etiopathology of preterm labor has been extensively investigated in recent years. Environmental and clinical risk factors include previous PTB history, low education and socioeconomic status, ethnicity, multiple pregnancies, short interval between pregnancies, maternal age (<16 or >36 years), obesity or low body mass index, hypertension, high maternal stress, uterine anomaly, or short cervix [138].

PTB is differentiated into spontaneous PTB, which follows preterm labor or premature rupture of membranes, and iatrogenic PTB, resulting from maternal or fetal conditions that contraindicate the continuation of pregnancy. Given that delivery is considered an inflammatory process, accumulating evidence shows that spontaneous PTB is associated with intrauterine infection or inflammation. Recent insights investigated the interaction between maternal microbiota/microbiome and spontaneous PTB. It has been noted that the host microbiota plays a crucial role in maternal and fetal immune interaction and influences various metabolic mechanisms and inflammatory processes, including PTB. Maintaining the microbiota in a more stable and protective pattern may help prevent spontaneous PTB. 

#### 3.3.1. Vaginal Microbiome Changes in Preterm Birth

In a full-term pregnancy, a “dynamic stability” of the vaginal microbiota is described as a higher concentration of Lactobacillus *species* from 20 weeks onward, thanks to increasing glycogen availability and a less complex and varied microflora [47].

This condition could be related to the lack of cyclic hormonal changes in pregnancy. *Lactobacilli* and low bacterial diversity are considered critical factors for achieving delivery at the end of gestation. Several studies also investigated the role of immune factors such as beta-defensin-2, which acts as a protective factor against spontaneous PTB. Beta defensin-2 modulates the risk of PTB regardless of the presence or absence of *Lactobacillus species*. Indeed, even when *Lactobacillus species* dominate the vaginal microbiota, low levels of beta-defensin-2 correlate with a higher rate of spontaneous PTB. However, the exact factors involved in regulating beta-defensin-2 levels still need to be determined.

When the balance between the maternal immune system and the vaginal microbiota fails, ascending microorganisms can colonize this site and lead to preterm labor. Reduced *Lactobacilli*, increased bacterial diversity, and low beta-defensin 2 are strongly related to a higher risk of PTB [139].

Vaginal colonization by *Lactobacillus* spp. cannot guarantee a full-term pregnancy, although it seems protective in early preterm births (less than 34 weeks). Clear evidence on the prevention of late preterm births (34–36 weeks) has yet to be demonstrated. *Lactobacillus crispatus* dominance characterizes full-term pregnancies, while the prevalence of *Lactobacillus iners* in the second trimester increases the risk of early spontaneous PTB. Indeed, *Lactobacillus iners* growth in ongoing pregnancy is a marker of vaginal microbiota instability. Unlike *Lactobacillus crispatus*, it can coexist in many cases with *Gardnerella* [138]. A study by Fettweis et al. demonstrated a correlation between the *L. crispatus/L. iners* ratio, vaginal inflammatory cytokine CXCL10 levels, and a higher risk of PTB [140].

Gram-negative bacteria dysbiosis can act as an inducer of preterm labor. An increase in pathogenic microorganisms such as *Gardnerella*, *Ureaplasma*, *Mycoplasma*, and *Prevotella* can lead to bacterial vaginosis, strongly related to chorioamnionitis, and doubles the risk of PTB, especially before 35 weeks. In addition, aerobic vaginitis, mainly caused by *Klebsiella*, *E. coli*, *Enterococci*, *Staphylococcus* spp., and *group B Streptococci* is related to spontaneous PTB. The vaginal administration of progesterone is widely used to prevent PTB, but this therapy does not seem to influence the vaginal microflora.

Vaginal swabs in the first trimester may act as a screening device to predict spontaneous PTB. The absence of *Lactobacilli* and polymicrobial vaginal colonization in early pregnancy swabs represent risk factors for PTB. A study conducted by Tabatabaei et al. also showed that, during the first trimester, a vaginal microbiome composed of *L. gasseri*, *L. jenseni*, *L. crispatus*, *L. acidophilus*, *L. iners*, *Ralstonia solanacearum*, *Bifidobacterium longum*, and *Bifidobacterium breve* might represent a lower risk of early spontaneous PTB compared to *Gardnerella vaginalis*, *Atopobium vaginae*, and *Veillonellaceae bacterium* [47].

The first trimester seems to be the ideal period to evaluate the vaginal microbiota since changes during this time could be related to long-term pregnancy outcomes.

#### 3.3.2. Oral and Gut Microbiome Changes in Preterm Birth

Periodontal pathogens and their products can reach the placenta and affect the fetal unit through blood circulation. Moreover, they might be involved in the development and progression of systemic inflammation. High periodontal pathogens during pregnancy have been associated with an increased risk for preterm delivery.

Notably, the number of *Porphyromonas gingivalis* was significantly higher in women with preterm birth [141]. Indeed, research by León et al. demonstrated the presence of microbial invasion in the amniotic cavity by *P. gingivalis* in pregnant women diagnosed with threatened premature labor [142].

Ye et al. determined the number of periodontopathic bacteria in the saliva, subgingival plaque, and placenta of patients with preterm labor. They found periodontopathic bacteria, such as *Aggregatibacter actinomycetemcomitans*, *Porphyromonas gingivalis*, *Tannerella forsythia*, *Treponema denticola*, *Fusobacterium nucleatum*, and *Prevotella intermedia*, that may access the placenta. The number of *F. nucleatum* and the detection frequency of *T. denticola* in placental samples were significantly higher in the preterm labor group [143].

Cassini et al. reported that periodontal pathogens might be present also in human urogenital tract microflora, probably derived from sexual habits. The most representative periodontopathic species found in the genital tract of the preterm group patients were *T. denticola*, *T. forsythia*, and *P. intermedia* [144].

Maternal gut microbiome dysbiosis also seems to be related to adverse pregnancy outcomes. However, the role of the maternal gut microbiome in triggering preterm birth remains poorly studied [145]. Yin et al. collected fecal samples from 41 women with threatened preterm labor and found a different gut microbiome composition than those who delivered at term. Opportunistic pathogens, such as *Porphyromonas*, *Streptococcus*, *Fusobacterium*, and *Veillonella*, were most represented, whereas *Coprococcus* and *Gemmiger* were significantly depleted in the preterm group. Interestingly, oral bacteria were the dominant community, suggesting that the oral cavity may represent an endogenous reservoir for the gut microbiome and that bacteria could migrate through the digestive tract [145]. 

**Figure 4:**
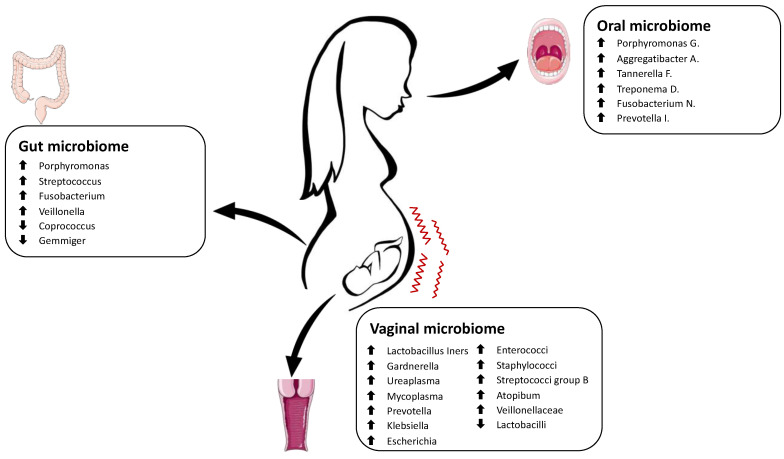
Schematic representation of the main alterations in the maternal microbiome in pregnancies complicated by preterm birth. The Figure was partly generated using Servier Medical Art, provided by Servier, licensed under a Creative Commons Attribution 3.0 unported license.

Figure 4 shows the main alterations in the maternal microbiome in complicated preterm birth pregnancies.

### 3.4. Recurrent Miscarriage

The recurrent miscarriage (RM) topic is getting more attention for its increasing incidence and negative impact on psychological health. It is defined by the European Society of Human Reproduction and Embryology (ESHRE) as two or more pregnancy losses before 24 gestational weeks (GA). It affects 1–3% of couples attempting to have a child [146].

Due to the need for clear etiologies and effective treatments, physicians still need to solve RM. The recognized causes of RM include chromosomal abnormalities, antiphospholipid syndrome, endocrinological disorders, thrombophilic disorders, uterine malformations, and infections [146,147,148]. However, the natural causes and pathogenesis remain unexplained in about half of the cases. Several adjuvant treatments have been considered in this group with idiopathic etiology of RM [148,149]. However, their effects on reducing pregnancy loss rates are still inconsistent [150,151].

The literature regarding microbiota alterations as a possible causative agent of RM is scarce and mainly focused on female genital tract microbial communities. The oral district has yet to be investigated, whereas only two studies analyzed the gut microbiota in unexplained RM patients. They suggest that butyrate-producing bacteria (*Roseburia*, *Prevotella*, and *Agathobacter*) may have an essential role in pregnant women as their amount was reduced in the gut of RM patients [152,153].

Most studies report that RM is associated with dysbiotic female reproductive tract microbiota, especially in the uterus [151,154,155,156,157,158]. Both in the vagina and the uterine cavity of RM patients, an increasing α-diversity and a lower abundance of *Lactobacilli* were detected compared to healthy women [154,155,156]. However, the critical microbiota alterations differ among locations, suggesting that different pathogenesis and treatment should be considered [156]. 

In the vaginal microbiota of RM patients, a significant increase in different bacteria is described: *Atopobium* [156,157], *Prevotella* [157,159], and *Gardnerella vaginalis* [154,159] are the most reported in the literature. Other research shows an increased abundance of *Pseudomonas* [158], *Streptococcus* [157], *Megasphaera*, and *Sneathia sanguinegens* [151]. No statistical differences were found in vaginal CSTs between RM and healthy controls [151].

The cervical microbiota is less explored in RM women and is similar to the vaginal microbiota. A lower abundance of *Lactobacillus* characterizes it including increasing levels of *Atopobium* and *Gardnerella vaginalis* [156,160].

The uterine cavity has long been thought to be sterile. Several researchers reported that endometrium might have a distinct microbiome influenced by bacterial ascension through the vagina [154]. In RM patients, the endometrial microbiome shows a lower abundance of *Lactobacillus crispatus* [154] and increased levels of *Gardnerella vaginalis* [154], *Acinetobacter*, *Anaerobacillus*, *Erysipelothrix*, *Bacillus*, and *Hydrogenophilus* [156]. Moreover, Liu et al. found a dramatic drop in IFN-γ and IL-6 levels inside the uterine cavity of RM women, suggesting an interaction between microbiota and the immunity system, even if negatively correlated (probably due to the small sample size of the study) [156]. This connection has also been suggested by Fan et al., as they found an increased expression of chemokine (CCL2, CCL3, CCL4, CCL5, and CCL8) in the villus tissues of women with RM [158].

In addition, the reduction in butyrate-producing bacteria in the gut microbiota of RM stresses this interaction because of their immunity maintenance and anti-inflammatory properties [152]. Liu et al. demonstrated an association between gut bacterial dysbiosis and a Th1/Th17-mediated proinflammatory state in miscarriage with unknown etiology [153]. Furthermore, the supplementary probiotic treatment seems to be helpful for couples with RM because of their capacity to improve the aberrant spermatozoa antigenicity [161].

Microbial dysbiosis is a risk factor for RM as the altered microbial environment (especially in the genital tract) may contribute to an adverse immunological response during fecundation, implantation, and placentation, probably by altering the “Th2 phenomenon” of pregnancy [153,162,163,164,165].

However, the underlying connections between the gut microbiota, the reproductive tract microbiota, the immunity system, and RM need to be further clarified. Further research should evaluate whether the prognosis of subsequent pregnancies can be assessed based on the microbiota profile and investigate the most appropriate treatment.

**Figure 5:**
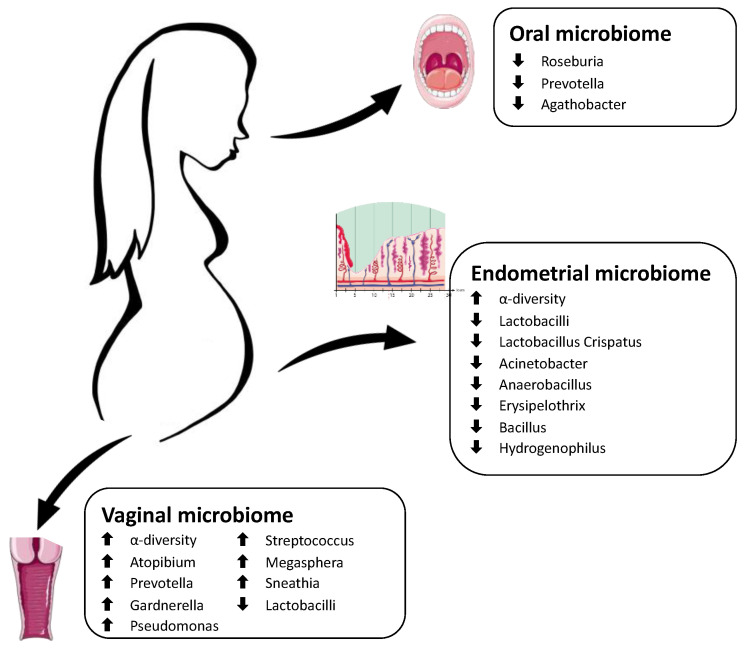
Schematic representation of the main alterations in the maternal microbiome in women suffering from recurrent miscarriage. The Figure was partly generated using Servier Medical Art, provided by Servier, licensed under a Creative Commons Attribution 3.0 unported license.

Figure 5 summarizes the main alterations in the maternal microbiome in pregnancies complicated by recurrent miscarriage. 

## 4. Therapies That Target the Microbiota in Pregnancy

As mentioned in the previous sections, a healthy microbiota is crucial for preventing diseases and maintaining overall health. It is well documented in the literature that the human microbiome has a pivotal role in maternal and child health outcomes [166]. Thus, diet, lifestyle, and intake of beneficial microbes profoundly impact the microbiota composition and function, but environmental exposure to microbes is also essential.

Diet is a critical factor in influencing health and aging courses since these effects are also mediated by the ability of nutrients to modulate the gut microbiota composition and, thus, metabolic function. However, not all diets are equivalent, and it is recognized that different dietary patterns exert distinct effects on the gut microbiota. For example, the Western diet (WD), characterized by high consumption of red meat, saturated fats, sugars, and generally processed foods, as well as a low intake of fibers, has profound effects on shaping the gut flora [167]. For this reason, adherence to a WD predisposes the onset of chronic-degenerative diseases, including some types of cancer [168]. On the other hand, a diet that is high in fiber and low in glycemic index carbohydrates, long-chain saturated fatty acids, animal protein (i.e., red and processed meat), and sugar, such as the Mediterranean diet (MD), can modulate the composition and functionality of human gut microbiota and reduce the risk of developing illness when compared to WD. Dietary fiber offers different substrates for fermentation reactions carried out by specific microorganisms to produce short-chain fatty acids (SCFAs) such as acetate, butyrate, and propionate, which in turn exert positive effects on gut health [168].

Once altered, prebiotics are foods or compounds used to rebalance the gut microbiota. Prebiotics must not be confused with probiotics, which are intended living microorganisms that, after administration in adequate quantities, offer beneficial effects on the host’s health [169]. Since both pro- and prebiotics are safe and well-tolerated even during pregnancy, their use is remarkably suggested. Together, they help facilitate the microbial balance of the gastrointestinal tract, increasing microbial diversity, improving intestinal barrier function, decreasing inflammation, and regulating insulin production [170]. In addition, thanks to these supplementations during pregnancy, the gut microbiota composition is modulated as well as an improvement in glucose and lipid metabolism [171]. Indeed, the mechanisms of action of probiotics and, therefore, their efficacy depend on the strains present in the preparation, thus explaining why many failed their treatments. Hence, a better understanding of the precise microbial modifications in each gynecological disease will offer insights into the choice of the accurate intervention instead of considering them as one size fits all [172]. 

Another important nutritional factor that has gained much attention in the last years is represented by Ω-3 fatty acids (FAs) and polyunsaturated FAs (PUFAs), primarily contained in fish meat, eggs, seafood, and vegetable oil. Administration of ω-3 fatty acids has been linked to amelioration in the composition and diversity of the gut microbiome. Furthermore, they possess anti-inflammatory properties as well as participate in neurodevelopment since they are fundamental in the brain lipid composition [173].

Physical activity (PA) is an additional modifiable lifestyle that modulates microbiota. PA, during pregnancy, has been proven to have beneficial effects on pregnant women, significantly improving several gestational complications [174] and promoting psychological well-being since endorphins are produced. However, how PA modulates the gut microbiota needs further investigation as it depends on different factors such as training intensity, environment, and diet [175]. 

Other factors that modify the human microbiota are represented by the indoor and outdoor environment. Environmental changes may lead to dysbiosis with important changes not only in the host but in its microbiota, inside and outside the body. This may result in an immunological imbalance leading to an inflammatory state, thus distressing different organs and systems [176].

All these aspects should be considered, as data from the literature showed a direct correlation between household and particulate pollution, the owning of a pet, and the modulation of the human microbiota [177].

## 5. Research Implications and Future Directions

The last few years have witnessed an explosion of information related to the role of microbiota changes in health and disease. One of the limitations of microbiome research is the different techniques used to identify the microbiome’s composition [178].

Such differences can create bias in data analysis and interpretation. For example, although there are growing studies in the literature, there is currently uncertain evidence for a placental microbiome. In this regard, the need for shared and standardized techniques regarding microbiome research is recommended.

The presence of a dynamic relationship between the commensal and host microbiota is today a relevant aspect of human physiology [179]. Likewise, an unbalanced microbiome is associated with specific clinical conditions. Microbial metabolites can modify cellular epigenetics in a healthy or harmful way (dysbiosis) [179]. Therefore, the presence of a dysbiotic state can affect the reproductive processes, including fertilization, implantation, placentation, and the immune system.

For what pregnancy disorders concern, maternal microbiome changes, its interaction with the immune system, and its involvement in the pathogenesis of obstetric complications remain poorly understood. Future studies evaluating the microbiome during pregnancy or after childbirth in the most common and impacting obstetric pathologies with standardized, uniform, and validated techniques could elucidate its role. Of interest could be the evaluation of the microbiome on tissues (in vivo or in vitro using organoids) rather than on biological fluids [180,181]. This additional evidence may provide insight into the need for a microbiome assessment in specific pregnancy disorders.

A better understanding of the maternal microbiota’s dysregulation may shed light on diagnostic or preventive measures to improve maternal and neonatal health. Future therapeutic strategies will likely be undertaken to modulate microbiota composition, among which we can mention the use of pro- and prebiotics and dietary changes. Nevertheless, further studies are warranted to provide specific tools that might be used to develop personalized therapies for disease prevention and tailored treatments.
